# An Improved Normalized Mutual Information Variable Selection Algorithm for Neural Network-Based Soft Sensors

**DOI:** 10.3390/s19245368

**Published:** 2019-12-05

**Authors:** Kai Sun, Pengxin Tian, Huanning Qi, Fengying Ma, Genke Yang

**Affiliations:** 1 School of Electrical Engineering and Automation, Qilu University of Technology (Shandong Academy of Sciences), Jinan 250353, Chinafengyma@163.com (F.M.); 2 Department of Automation, Shanghai Jiao Tong University, Shanghai 200240, China; 3 Ningbo Artificial Intelligence Institute, Shanghai Jiao Tong University, Ningbo 315000, China

**Keywords:** soft sensor, variable selection, neural network, mutual information, tabu search

## Abstract

In this paper, normalized mutual information feature selection (NMIFS) and tabu search (TS) are integrated to develop a new variable selection algorithm for soft sensors. NMIFS is applied to select influential variables contributing to the output variable and avoids selecting redundant variables by calculating mutual information (MI). A TS based strategy is designed to prevent NMIFS from falling into a local optimal solution. The proposed algorithm performs the variable selection by combining the entropy information and MI and validating error information of artificial neural networks (ANNs); therefore, it has advantages over previous MI-based variable selection algorithms. Several simulation datasets with different scales, correlations and noise parameters are implemented to demonstrate the performance of the proposed algorithm. A set of actual production data from a power plant is also used to check the performance of these algorithms. The experiments showed that the developed variable selection algorithm presents better model accuracy with fewer selected variables, compared with other state-of-the-art methods. The application of this algorithm to soft sensors can achieve reliable results.

## 1. Introduction

In real industrial processes, many variables cannot be measured at all or frequently enough with traditional instrumentation, owing to the technological or economical constraints of insufficient space, poor environmental conditions, extreme operating conditions, etc. Soft sensors provide valuable solutions for estimating these process variables through inference modeling with other easily measured variables. Paulsson, et al. [1] applied a soft sensor to a microbial recombinant protein production process in a bioreactor by exploiting bio-calorimetric methodology and obtained better results compared to other researchers. Chen, et al. [2] proposed a local soft-sensing method based on real-time correlation to predict silicon content, and better prediction performance of the method was validated on the online silicon content prediction. Kaneko, et al. [3] applied a soft sensor to the fault detection of chemical plants in order to accurately detect faults in the production process. Ahmed, et al. [4] successfully predicted the change of melt index during the production of high-density polyethylene using soft sensor technology. Wang, et al. [5] developed and applied a soft sensor to a refining process for quality prediction and achieved a high prediction accuracy. Xing, et al. [6] proposed a soft sensor for silicon content in industrial blast furnaces based on bagging local semi-supervised models (BLSM), the application results showed that BLSM had better prediction performance compared to other supervised soft sensors. Yuan, et al. [7] developed a novel variable-wise weighted stacked auto encoder (VW-SAE) for a soft sensor, and industrial data-based experiments showed that the proposed VW-SAE could present better prediction performance than traditional algorithms. Mambou, et al. [8,9] have also done some valuable research in this field.

In fact, most industrial processes are highly non-linear. Artificial neural networks (ANNs) are widely used in soft sensor technology because of their strong nonlinear fitting ability. Feil, et al. [10] presented a soft sensor for melt index by using a semi-mechanistic modeling approach; the ANN-based modeling approach can be efficiently used for on-line state estimation. Ko, et al. [11] used a soft sensor based on ANNs to predict the grain size of ore, and the resulting model can accurately measure the grain size distribution in industrial production in real time. Shakil, et al. [12] developed a soft sensor for measuring gas emissions during industrial boiler combustion using a dynamic ANN, and measurement results with a high accuracy were obtained. Bo, et al. [13] constructed an adaptive soft sensor instrument based on ANN technology and applied it to an advanced control system, the control system realized the quality closed-loop control very well. Jiesheng, et al. [14] used an ANN soft sensor model to predict the concentrate grade. Simulations show that the model has better generalization results and prediction accuracy.

The rapid development of distributed control systems (DCS) provides us with a huge amount of industrial information [15,16]. The big data also presents us with another problem in non-linear soft sensing: there are many redundant input variables in the process. If too many input variables are introduced in the training process of the ANN, the data dimensions increase and more computational power is required. At the same time, introducing variables that are unrelated to the prediction results will increase the noise in the dataset and reduce the prediction accuracy of the ANN. Therefore, the study of effective variable selection algorithms for soft sensors has attracted interest from many researchers. Variable selection algorithms can be broadly divided into two categories: wrapper and filter algorithms. The wrapper algorithms essentially treat the selection of inputs as an optimization of the model structure. They compare and evaluate either all or a subset of the possible input variable sets and select the set that yields optimal performance [17,18]. Wrapper algorithms utilize the performance of machine learning to evaluate the goodness of feature subset and, therefore, have great chance of leading to good prediction performances. Due to this advantage, the wrapper algorithms are widely studied and applied [19,20,21,22,23,24]. In spite of the performance, wrappers can be very computationally expensive since many predictive models with different feature subsets have to be built. Moreover, the results of the wrapper strategy lack generality as their use is limited to a specific regression model.

The filter algorithms utilize a statistical measure of the degree of dependence between the input and output variables as the criterion for variable selection. Compared to the model-based wrapper algorithm, the filter algorithms are model free and have less computational complexity. Among them, mutual information (MI) is a very suitable measure of dependence for variable selection during ANN development, since it is an arbitrary measure and makes no assumption regarding the structure of the dependence between variables. For a modeling problem, the ultimate objective is to reduce as much as possible an error criterion; the most frequently used is the mean squared error (MSE). Frénay et al. [25] demonstrated that that MI was an adequate criterion for feature selection with respect to the MSE, when the estimation error was identically distributed for any input variables with a uniform, Laplacian or Gaussian distribution. The MI also has been found to be robust due to its insensitivity to noise and data transformations [26,27]. Therefore, many MI-based feature/variable selection algorithms have been proposed. Hanchuan, et al. [28] developed a minimal redundancy maximal relevance (mRMR) criterion to minimize redundancy, and made use of a series of intuitive measures of relevance and redundancy to select meaningful features. Estévez, et al. [29] proposed an enhanced version of mutual information feature selection (MIFS) and mRMR that introduced the normalized mutual information (NMI) as a measure of redundancy. The proposed normalized mutual information feature selection (NMIFS) algorithm showed better performance than MIFS and mRMR, by compensating for the MI bias toward multivalued features and restricting its values to the range [0, 1]. Bennasar, et al. [30] gave a normalized joint mutual information maximisation (NJMIM) for feature selection, which is based on the “maximum of the minimum” criterion. The results showed that the NJMIM method outperforms the other methods on several tested public datasets. Novovicová, et al. [31] designed a sequential forward feature selection method based on conditional mutual information called CMIFS to find a subset of features that are most relevant to the classification task. The feature selection algorithm has better classification accuracy on high dimensional Reuters textual data.

However, the NMIFS also has some disadvantages. One of them is that the execution of the variable selection only depends on the data characteristics and has nothing to do with the structure and model accuracy of the ANN. Another disadvantage is that the algorithm is essentially a greedy search; it only deletes one variable at every iteration. This mechanism will bring about an inherent drawback, in that the solution tends to fall into a local optimum.

The tabu search (TS) algorithm can avoid local optima by extending local neighborhood searches. The research of Zhang, et al. [32] showed that TS is a promising tool for variable selection due to the quality of its obtained variable subset and its computational efficiency. Pachecoaab, J. [33] proposed a TS method that could find a smaller subset from a set of variables, to effectively classify cases. Osman, I. H. [34] used TS for the vehicle routing problem; the new method provided a set of lowest-cost delivery routes and achieved satisfactory results. Lin, et al. [35] applied TS to an economic dispatch problem, and results showed that the proposed algorithm can provide accurate solutions with reasonable performance.

In this paper, an improved NMIFS for soft sensors is proposed. To the best of our knowledge, this is the first implementation developing a neural network-based soft sensor by combining NMIFS with TS. The contributions of this paper are as follows:

(1) The proposed algorithm performs variable selection by combining the NMI and training accuracy of neural networks.
(2) TS is used as an auxiliary optimization method, and it effectively prevents the proposed algorithm from falling into local optimums.
(3) The developed soft sensor is applied to predict the sulfur dioxide (SO_2_) emissions of flue gas in a practical power plant, and it has better performance than other algorithms.

The paper is organized as follows. Section 2 reviews the theories of NMIFS and ANN. Section 3 presents the design and analysis of the proposed algorithm. Section 4 gives the numerical simulation results of two artificial dataset examples. Section 5 shows that the soft sensor based on NMI and TS (NMI-TS) achieved an accurate measurement of flue gas SO_2_ concentration. Finally, some concluding remarks are given in Section 6.

## 2. Theoretical Overview

### 2.1. Input Variable Selection Techniques

In the training processes of ANNs, redundant candidate variables tend to complicate the model, reduce the modeling accuracy, and can even lead to the over-fitting phenomenon. The purpose of the input variable selection (IVS) technique is to correctly select the relative variables from the candidate variable set **C** that contains all the potential input variables of the process. In the variable selection process, variables that contribute less or even have negative effects to the model accuracy will be excluded.

In general, the IVS can be implemented in three ways: (1) sequential forward selection, starting from a null set and selecting a variable into target set at every iteration until there is no improvement in the prediction accuracy of the model; (2) sequential backward selection, starting from set **C** and deleting a variable at a time until the termination rule is met; (3) global optimization, finding the best variable set among all the possible schemes with optimization algorithms.

Mathematically, if there are **m** input variables in **C**, there will be totally (2^m^ − 1) subsets in the selection result set [36]. Therefore, it is very difficult to find the best solution if the number of input variables is large. Based on this consideration, it is necessary to find a statistical metric by which to scale the degree of dependency between the input and output variables. Then, we can preliminarily select the input variable before modeling with ANNs. This statistical pre-processing input variables simplifies the complexity of modeling and can provide resulting solutions that have wider adaptability to different ANN structures. However, the performance of the IVS algorithm is highly dependent on the statistical metrics used.

During the development of IVS algorithms for ANNs, MI has been found to be a valuable evaluation criterion, as it is an arbitrary measure and makes no assumption regarding the structure of the inter-variable dependencies.

### 2.2. Mutual Information (MI)

**Figure 1:**
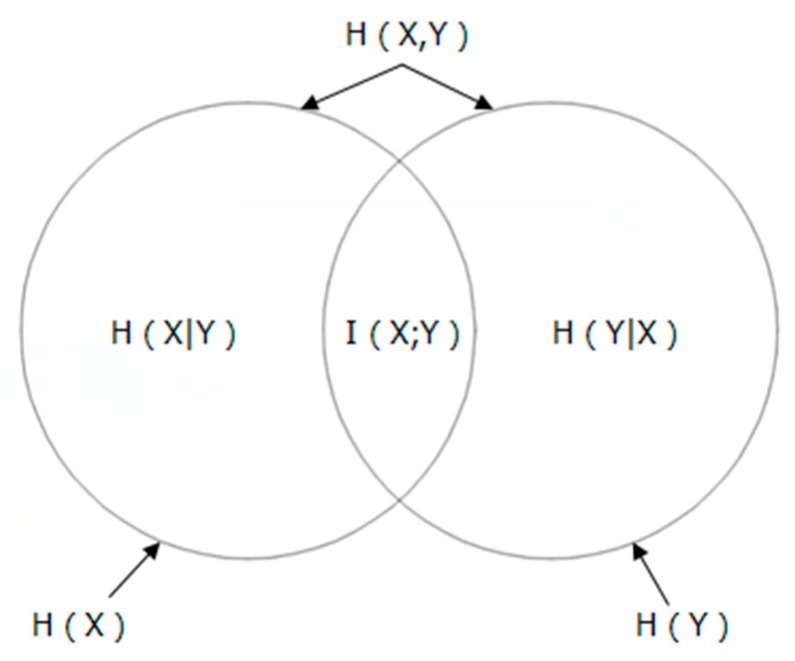
Relationship between entropy and mutual information.

Suppose that Y is a random output variable there will be some uncertainty in the observation value y∈Y. The uncertainty is defined on the basis of Shannon entropy, H. Suppose X is a random input variable and Y is output variable dependent on X, the mutual observation of (X,Y) will lower uncertainty as the knowledge of X allows inference toward Y. For a continuous variable, the definition of MI can be expressed as follows [34]:

(1) $$I\left(X;Y\right)=\iint p(x,y)\log\frac{p\left(x,y\right)}{p\left(x\right)p\left(y\right)}\mathrm{dxdy}$$

where “p(x, y)” is the joint probability density function (PDF), and “p(x)” and “p(y)” are the marginal PDFs of X and Y, respectively. The entropy of X and Y and its relationship to their MI is shown in Figure 1.

MI has three primary properties:

1. Symmetry: I(X;Y) = I (Y;X).The information extracted from Y about X is of the same amount as the information extracted from X about Y. The only difference between them is the angle of the observer.
2. Positive: I(X;Y) ≥ 0. For all extracting information, the worst case scenario is that there is no information, that is I(X;Y) = 0
3. Extremum: I(X;Y) ≤ H(X), I(Y;X) ≤ H(Y). The amount of information extracted from one event about another is at most equal to the entropy of the other event, and does not exceed the amount of information contained in this other event itself.

The I(X;Y) measures the dependence of X and Y and provides useful information for variable selection. Therefore, how to compute I(X;Y) is very pivotal to MI-based variable selection algorithms. The difficulty of calculating I(X;Y) is that the real functional form of the PDF in (1) is unknown in real world. To solve the difficulty, many approximate estimation algorithms of MI have been widely studied to resolve PDFs. For instance, a kernel density estimation (KDE) exerts a basis function to every point of the feature data and has good computational quality [28]. Then, the PDF approximation can be achieved by adopting an envelope of all the basic functions exerted on each point. K-nearest neighbor (KNN) [37,38] is also an effective method to solve this problem. Despite superior approximation results, these kinds of algorithms have significantly high computational load, especially for large-scale problems. Histogram methods [39] present an alternative solution with acceptable accuracy and conspicuously higher efficiency than KDE methods.

The computational results fluctuate a lot when MI is used to actual production problems. For this reason, it can hardly be taken as an indicator to compare the similarity between different variables. A new method is introduced to normalize MI in which the entropy is applied as the denominator to adjust the value of MI within [0, 1]. The expression is shown as:

(2) $$N\left(X,Y\right)=2\frac{I\left(X,Y\right)}{H\left(X\right)+H\left(Y\right)}$$

Following that, NMI can be effectively applied to assess the similarity between the input variables and the target variables.

Inspired by Battiti and Hanchuan [26,28], the evaluation criterion whether a variable is selected is designed as follows:

(3) $$B_{j}=I\left(Y;\mathrm{pj}\right)-\xi \frac{1}{k}\overset{k}{\underset{i=1}{\sum }}I\left(a_{i};p_{j}\right)$$

where Y is the output variable, p_j_ is the pending variable, $a_{i}$ is the selected variable, and ξ is a constant that is used to control the ratio of redundant items in $B_{j}$. The parameter ξ is used as a factor for controlling the redundancy penalization among single features and has a great influence on FS. The values of ξ are different according to the sample size, complexity and number of variables, and are usually determined experimentally based on the specific problem.

### 2.3. Tabu Search

TS is an intelligent optimization algorithm that extends local neighborhood searches. The most important idea of TS is to mark the local optimal solution that has been reached and try to avoid these objects in the further iterative search, to ensure the exploration of different effective search approaches.

The solution quality of greedy search algorithm depends on the starting point and neighborhood structure, and it is very easy to fall into a local optimum. To obtain better solutions, researchers usually expand or change neighborhood structure, or start searching from multiple starting points. However, these strategies still cannot guarantee the global optimum of the solution due to the nature of their “greed”.

Unlike a greedy search, TS has the advantage of being able to escape from a local optimum by accepting inferior solutions. The implementation of TS involves the design of neighborhood, tabu list, tabu length, amnesty rule, termination rule and constitutes a loop with them in the search process.

At the beginning of TS, an initial solution is generated randomly or by some heuristic algorithm. Then, the quality of all solutions in the neighborhood is evaluated according to the fitness function. For a minimization problem, the solution that has smallest objective value has largest fitness function and will be selected as the solution of next iteration. The tabu list is a short-term memory of the specific changes of recent moves within the neighborhood, and can be used to preventing future moves from undoing those changes. Tabu length is the length of the tabu list. The amnesty rule is applied to avoid lost superior solution. If a solution in the tabu list is obviously better than other solutions, it can be taken as the solution of the current iteration ignoring the tabu rules. The termination rule is to stop the search process when the number of iterations reaching the limit or the obtained solution satisfying the preset accuracy.

Define the current solution as x, neighborhood as N(x), fitness function as C(x), tabu list as T. The algorithm flow of TS is described as follows:

(4) C(x*)=max{C(x),x∈N(x)\T}

where X* is the optimal solution that is not written into the tabu list in the neighborhood. The algorithm flow as follows:

Step 1 Generate an initial solution x ∈ X, set x* = x, T = ∅, define amnesty rule as A(x) = C(x*), iterations k = 0.
Step 2 k = k + 1, if k > MK (MK is the maximum number of iterations), Stop the search.
Step 3 C($x_{L}$) max{C(x),x∈N(x)}, if A(x) < C($x_{L}$), set x = $x_{L}$, jump to Step 5.
Step 4 C($x_{K}$) max{C(x),x∈N(x)\T}, set x = $x_{K}$.
Step 5 If C(x) < C(x*), set x = x*, C(x*) = C(x), A(x) = C(x*).
Step 6 Add x* to tabu list, release the solution that reaches the tabu length and return to Step 2

TS is widely used for combination optimization problems and has superiority in global search [40,41]. In this paper, we adopted the search mechanism of TS and incorporated it with NMIFS to develop a new variable selection method for soft sensors.

## 3. Design and Analysis of Algorithms

### 3.1. Proposed Methods for Feature Selection

Equation (4) not only contains MI between p_j_ and Y, but also takes MI between p_j_ and selected variables as punishment. This means that there should be a high degree of dependency between p_j_ and the output variables, and p_j_ should also have a lower dependency on the variables in **A**. In this way, variables that have less impact on the prediction accuracy, or redundant input variables, can be prevented from being added to **A**. It avoids increasing the size of the model and reduces the data processing time required in training the ANN. At the same time, the model input noise can be reduced to improve the accuracy of the model.

If the input variables are selected as described above, a termination criterion needs to be set for the algorithm. First, for each variable p_j_ ∈ **P**, compute $B_{j}$, then find the p_j_ that maximizes $B_{j}$; set **P** ← **P**\{p_j_}; set **A** ← {p_j_}, and the algorithm stops when all $B_{j}$ are less than a scalar quantity. The scalar is a parameter that needs to be input manually, and different values are required for different IVS problems. Because this algorithm is a greedy search algorithm, it will produce a local optimal solution. To ensure the exploration of different effective searches, we used a TS algorithm.

In the process of programming the algorithm, we can set the tabu list as a “tube”; at each iteration, the element that needs to be put into the tabu table is packed into a ““ball”, and the number of balls that the tube can accommodate is called tabu length. Then we press the ball into the tube inlet; if a ball emerges from the outlet of the tube, it indicates that the elements have reached the tabu length. If there are no balls appearing from the outlet of the tube, it means that the elements in the tabu list have not reached the tabu length. The size of the neighborhood is set to N and the tabu list to S. The algorithm flow as follows:

Step 1 Evaluate I(Y; p_j_); for each variable in P, Select the variable corresponding to the biggest I(Y; p_j_) as the starting point.
Step 2 If P≠∅ or S≠∅, Stop the search.
Step 3 The variables satisfying the tabu length were taken from S and added to P.
Step 4 Evaluate $B_{j}$ for each variable in P.
Step 5 Sort the variables in P by $B_{j}$, select the first N variables and put them in neighborhood.
Step 6 Evaluate $\lambda_{n}$ (fitness value) for each variable in the neighborhood.
Step 7 Select the variable corresponding to the smallest $\lambda_{n}$ as the optimal solution of this iteration
Step 8 Put the other variables in S and return to Step 2.

The pseudo-code of the algorithm is shown in Algorithm 1. The algorithm can be regarded as a combination of local search (LS) algorithm and TS. The LS algorithm is a sequential forward selection approach that consists of two loops. The outer loop performs variable selection by choosing the variables with the highest fitness at every step, where the fitness of a variable is defined by its NMI. Then the algorithm takes the variables selected by the outer loop and enters them into the inner one. The inner loop performs TS by selecting the variable with the lowest fitness at every step, then putting the other variables into the tabu list.

The algorithm proceeds until all variables are selected into **A**, then the algorithm can obtain a “path” according to the order in which variables are selected into **A**. Along this known path, I (I = 1, 2, 3, …, i, i is the length of **A**) variables are selected to train the ANN at each iteration, and the cross-validation (CV) MSE is used to evaluate the modelling accuracy and select the optimal solution.


**Algorithm 1: Pseudo-code of NMI-TS algorithm**

**Input: dataset {P, Y}**

**Results: new dataset A after selection**
While P ≠ ∅ or S ≠ ∅  release the solution that reaches the tabu length;   j = 1;  While j <= the length of P    $B_{j}=I\left(Y;p_{j}\right)-\xi \frac{1}{k}\sum_{i=1}^{k}I\left(a_{i};p_{j}\right);$    J = j + 1;  end  Sort the variables in P by B_j_ from largest to smallest and select the first N variables, put them in set M;  N = 1;  While n <= N    Evaluate λ_n_ for nth variable in M; (by using **Algorithm 2**)  n = n + 1;  end  Select the variable corresponding to the smallest λ_n_ and add it to A, Put the other variables in S; End

### 3.2. Subroutine of Cross-Validation (CV)

CV is a common method used in machine learning to build models and validate model parameters. To achieve the *k*-fold CV, we need to reserve a single sub-dataset (validation set) at the start and use the remaining *k* − 1 sub-datasets (training set) to train a new ANN, then obtain MSE by validating the reserved sub-dataset with the new ANN. The *k*- fold CV process is repeated *k* times with each of the *k* sub-datasets used only once as the validation set, and then produce a single estimation $\lambda_{n}$ with the averaged results.

Algorithm 2 presents a detailed subroutine of CV, in which the MSE is used as the evaluation index in selecting variables. The *k*-fold CV procedure is implemented by the loop: (1) reserves a single sub-dataset, (2) uses the remaining *k* − 1 sub-datasets to retrain a new ANN and (3) obtains the MSE by validating the reserved sub-dataset with the retrained ANN.


**Algorithm 2: Subroutine of CV**
**Input: dataset {A, Y}** **Results: fitness value λ_n_** Divide{A,Y}into k disjoint sub-datasets{A_1_,Y_1_},{A_2_,Y_2_},{A_3_,Y_3_},……,{A_k_,Y_k_}; For p = 1:k  Set A as the input variable, Y as the output variable;  Train a new ANN with dataset {A,Y}={A,Y}-{A_k_,Y_k_};  Validate the dataset {A_k_,Y_k_} on the new ANN and the MSE denoted as MSE_p_; End $\lambda_{n}=\frac{1}{k}\sum_{p=1}^{k}{\mathrm{MSE}}_{p};$

## 4. Numerical Examples

### 4.1. Experimental Setting

All the algorithms in the paper are tested in the same experiment setting. The programs used for conducting the numerical experiments was coded in MATLAB 2016 and run on a windows 8.1 operating system. They used the same ANN structure that has one hidden layer with the hyperbolic tangent activation function and an output layer with the linear activation function. The ANN are trained using the standard back-propagation algorithm [42]. The number of hidden nodes are determined by trial experiments.

In the experiments of Section 4, 80% of the dataset is used for modeling, and 20% is used to test the accuracy of the model. The simulation results are obtained with the following definitions:

(1) Model size (M.S): The number of input variables selected by the algorithm.
(2) MSE: During the whole modeling process, part of the data is used for modeling, and the other part is used to verify the accuracy of the model. We recorded MSE between the predicted and desired output to evaluate the prediction accuracy of the model.
(3) Coefficient of determination $\left(R^{2}\right)$: the square of the sample correlation coefficients between the actual and predicted values.
(4) Positive wrong selection (WS+): The ratio of unselected valid variables to all valid variables and the number of unselected valid variables.
(5) Negative wrong selection (WS−):The ratio and number of irrelevant variables in the results.

### 4.2. Case 1

In the example, a nonlinear dataset containing a pool of candidate variables was generated to validate the performance of the methods. The true non-linear model [24] was:

(5) $$y=\{\begin{array}{c} \sqrt{X\cdot \beta -0.5}+\varepsilon \;\;X\cdot \beta \geq 0.5\\ e^{X\cdot \beta +0.5}+\varepsilon \;\;\;X\cdot \beta \leq 0.5 \end{array}$$

The input dataset X was generated from a multivariate normal distribution with covariance matrix ∑. The covariance between two different variables (columns) was:

(6) $$\sum_{i,j}{=\rho }^{\left|i-j\right|},\forall i\neq j$$

and the coefficient ρ of the covariance matrix was set to 0.2, meaning that multi-collinearity was introduced, ε is white Gaussian noise, where β = [3.0, 1.5, 2.0, 4.0, 0.5, 1.3, −2.6, −3.5, −5.1, 2.0]^T^ which was taken from [43]. Set the dataset $X_{1}\in R^{2000\times 10}$ was the set of relevant variables, $X_{2}\in R^{2000\times 10}$ was the set of irrelevant variables, then the input dataset X = {$X_{1},\;X_{2}$}.

In order to compare the performance of the proposed algorithm, some state-of-art MI-based variable selection algorithms such as NMIFS, and CMIFS [31] are applied to the numerical dataset. Moreover, an advanced wrapper algorithm that combines nonnegative garrote with ANN, called NNG-ANN [24], is also applied in the experiments. Table 1 presents the performance of the simulation results with different algorithms. It can be seen from the table that NMI-TS has the best MSE and $R^{2}$ with the fewest variable selected. The WS+ is zero, meaning that the algorithm selects all the relative input variables. The WS- is one, meaning that only one irrelative variable is selected in the final model. The results show that the NMI-TS is superior in variable selection accuracy to other algorithms.

**Figure 2:**
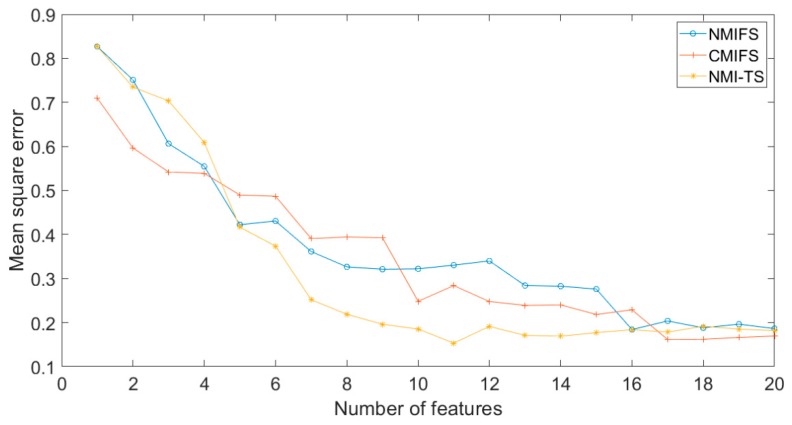
The curves of mean squared error (MSE) number of features of Case 1 dataset with mutual information (MI)-based algorithms.

The curves of MSE to the number of features of this dataset with MI-based algorithms are shown in Figure 2. Note that the characteristic of NNG-ANN is to give the best solution at a time instead of presenting a solution path. Therefore, the curves of NNG-ANN are not presented in this type of figures. It can be seen from the figure that the MSE reaches the best result at 11 features. At those points fewer than 11 features, the performance deteriorates drastically because of the lack of necessary input information. At the right of the best point, MSE slowly deteriorates due to the introduction of redundant information as the number of features increases. Moreover, the other algorithms converge more slowly to reach a better point than NMI-TS.

To further verify the performance of the algorithm, we conducted the following experiments on the above dataset

(1) Sample size

**Figure 3:**
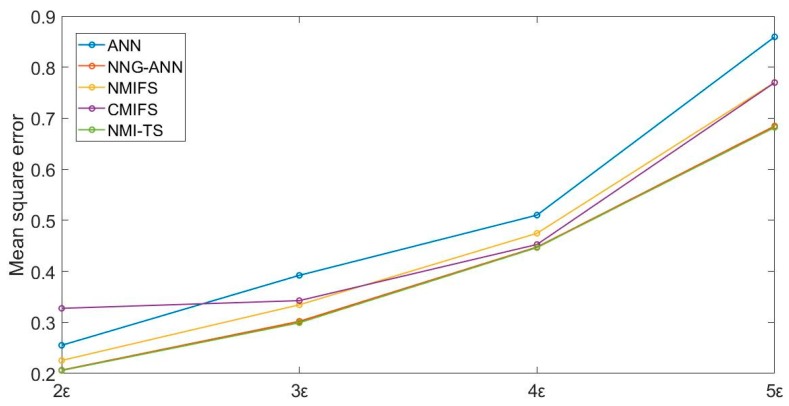
The simulation experiment with different sample size.

We scale the sample size down to 200–800 and then conducted four experiments using MSE as the evaluation criterion. The results are shown in Figure 3.

(2) Correlation

**Figure 4:**
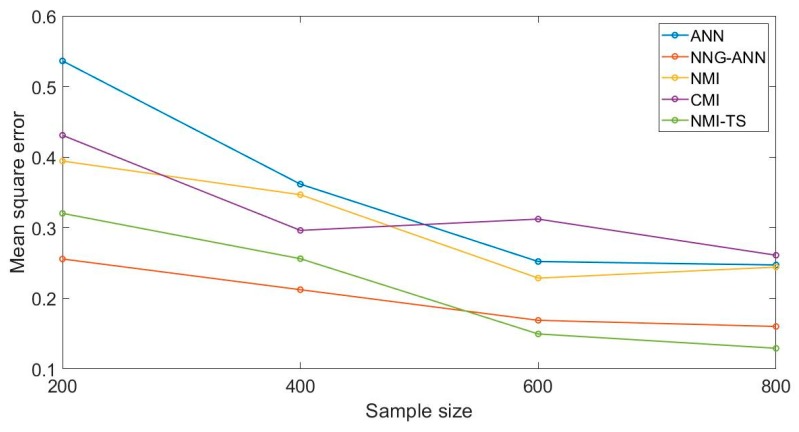
The simulation experiment with different correlation.

The correlation between the input variables ρ increased from 0.2 to 0.8, and we then conducted experiments with MSE as the evaluation criterion. Results are shown in Figure 4.

(3) Noise intensity

**Figure 5:**
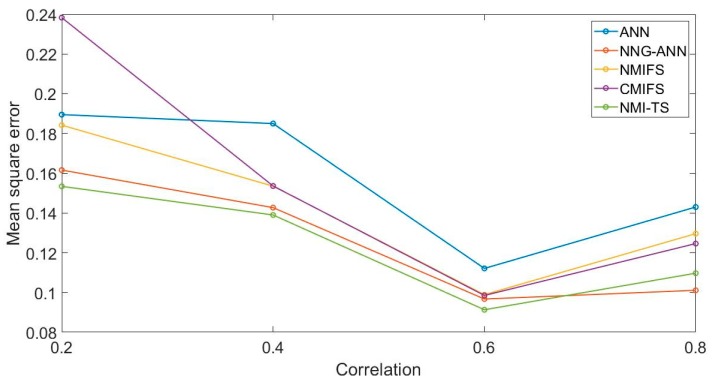
The simulation experiment with different noise.

ε in Equation (5) was increased and we designed the experiments with MSE as the evaluation criterion. The results are shown in Figure 5.

Sample size, correlation, and noise intensity all affect the prediction accuracy of the algorithm, especially the modeling accuracy of the ANN. Because MI is used as the evaluation criterion, CMIFS NMIFS and NMI-TS are less affected. Although the MSE of CMIFS NMIFS and NMI-TS are essentially the same in some experiments, NMI-TS usually presents a better model accuracy with fewer variables selected. This algorithm can solve the problem of strong coupling between input variables. It is worth noting that the accuracy of the proposed algorithm has certain dependence the amount of training samples. If the training sample is too small, the sparse distribution of experimental data will reduce the reliability of histogram method. Then, the PDFs between variables cannot be effectively calculated, which will lead to the deterioration of the algorithm performance. Although the utilization of KDE or KNN can improve this situation, the cost of operation is also greatly increased.

### 4.3. Case 2

**Figure 6:**
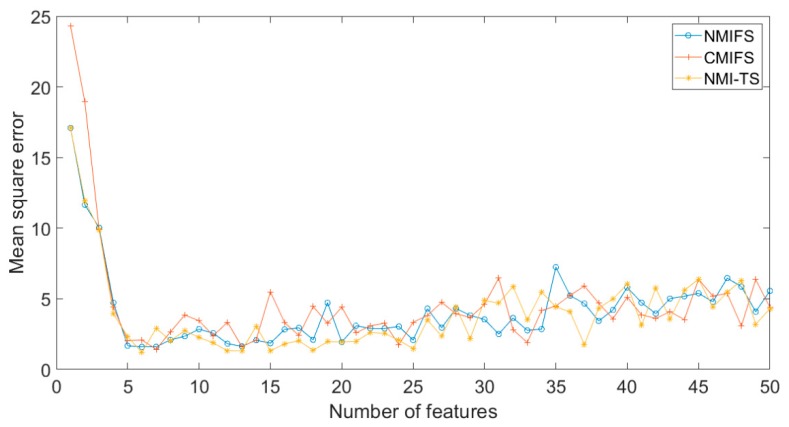
The curves of MSE number of features of Case 2 dataset with MI-based algorithms.

In order to further validate the proposed algorithm, a benchmark dataset that was first proposed by Friedman, J. H. [44] is applied in our paper. The dataset used in the paper has five input variables relevant to the output variable, which is calculated by

(7) $$y=10\sin\left({\pi x}_{1}x_{2}\right)+{20(x}_{3}-{0.5)}^{2}+{10x}_{4}+{5x}_{5}+\varepsilon$$

where {$x_{1}$, $x_{2}$, $x_{3}$, $x_{4}$, $x_{5}$} are relevant input variables and ε is white Gaussian noise. In addition, this dataset also contains 45 input variables that are irrelevant to the output variable. The role of the variable selection algorithms is to select {$x_{1}$, $x_{2}$, $x_{3}$, $x_{4}$, $x_{5}$} from the total of 50 variables. However, the irrelevant variables have different degrees of correlation to {$x_{1}$, $x_{2}$, $x_{3}$, $x_{4}$, $x_{5}$} ranging from 0.3 to 0.9, which increases the difficulty of variable selection. Table 2 presents the performance of the simulation results on the dataset with different algorithms. As we can see, the NMI-TS has smaller MS and better MSE than other approaches. The curves of MSE to the number of features of this dataset with MI-based algorithms are shown in Figure 6. The figure shows that the NMI-TS converges fast to the best solution, while the other methods require more variables to converge.

## 5. Application to Actual Desulfurization Process

### 5.1. Desulfurization Technology of the Power Plant

The power plant providing the data used in this article operates a limestone-gypsum wet flue-gas desulfurization technology, which uses lime or limestone to react with SO_2_ and absorb it. The chemical reaction principles are:

(8) $${\mathrm{SO}}_{2}+\mathrm{CaO}+\frac{1}{2}H_{2}O\to {\mathrm{CaSO}}_{3}\cdot \frac{1}{2}H_{2}O$$

(9) $${\mathrm{SO}}_{2}{+\mathrm{CaCO}}_{3}+\frac{1}{2}H_{2}O\to {\mathrm{CaSO}}_{3}\cdot \frac{1}{2}H_{2}{O+\mathrm{CO}}_{2}$$

The flue gas containing SO_2_ moves up from the bottom of the absorption tower, it encounters the spray layer of alkaline spray and reacts with it to form liquid drops. Tiny liquid drops fall into the slurry pool, where the oxidation of CaSO_3_ and the crystallization of CaSO_3_·1/2H_2_O are completed; finally, gypsum is formed at the bottom of the absorption tower.

**Figure 7:**
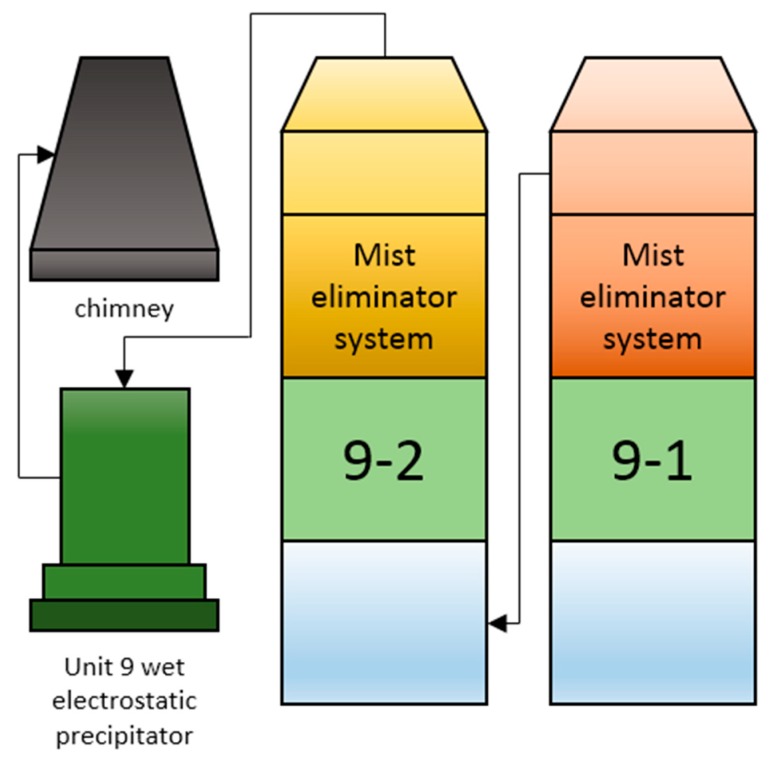
The flow direction of flue gas in the flue-gas system.

The desulfurization process and related parameters are mainly taken from unit 9 of the power plant. The main feature of this unit is that it adopts the structure of a double absorption tower. Compared with the design of the single absorption tower, this structure can be used for secondary treatment of the discharged flue gas, and can remove SO_2_ in the flue gas more effectively. Figure 7 shows the flow direction of flue gas in the flue-gas system.

In the entire desulfurization process, the untreated flue gas is first be introduced into the two-stage absorption tower, and once desulfurized it is introduced into the chimney through a high-pressure dust catcher.

**Figure 8:**
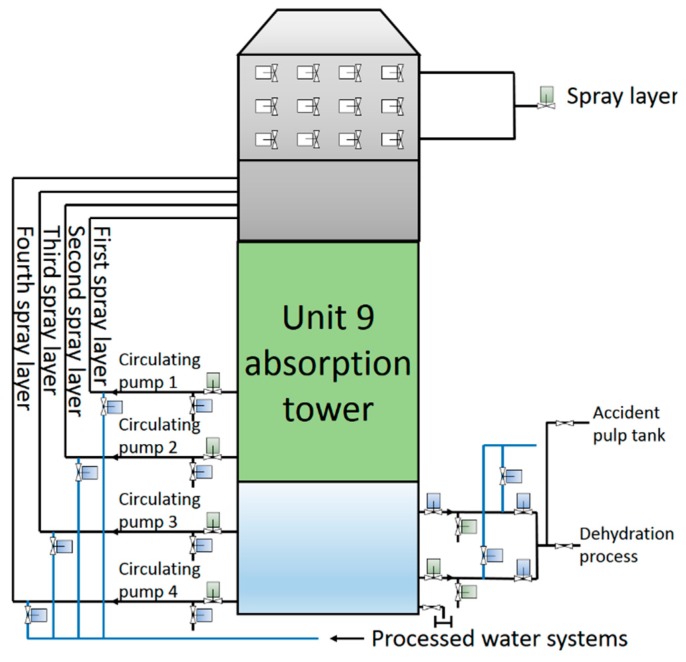
The structure of absorption tower.

A fan pressurizes the flue gas and sends it to the bottom of the No.9-1 absorption tower, but before this happens it needs to be cooled down. The reason for this is that, within a certain range, lower temperatures are more conducive to the absorption of SO_2_ in the flue gas. The flue gas rises from the bottom of the first absorption tower and reaches the top after being treated, and then it is sent to the bottom of the secondary absorption tower for the second treatment. With wet flue-gas desulfurization technology it is easy to produce “fog” with particle sizes of 10–60 microns in the absorption tower. Because the “fog” produced by this process contains SO_2_, the gas inside the tower is defogged through a mist eliminator before leaving the absorber. To prevent the liquid in the slurry tank of the absorber from pouring back into the flue-gas inlet, it is necessary to set an overflow port at the upper part of the slurry pool of the first stage absorber. The overflow port of the secondary absorber will overflow the slurry to the first stage absorber when a high liquid level is reached. Due to the large volume of the slurry pool, if the liquid within it remains stationary for a long time, solid precipitation will be generated. To prevent the depositing of solids in the slurry pool, pulsed suspension pumps are used to stir the slurry. Given that oxidation and crystallization processes are required in the slurry tank, an oxidation air blower is used to feed the air to the tower through an aeration device arranged in the slurry tank. Because there is less gypsum produced by the slurry pool in the secondary absorber, slurry is directly fed into the slurry pool of the first stage absorber by the gypsum swirl pump, then the oxidation and crystallization process is carried out. Apart from the different assembly of overflow ports and oxidation air blowers, the structure of the two absorption towers is largely the same, as shown in Figure 8.

### 5.2. Expriment’s Design and Results

In the desulfurization process, the untreated flue gas is first introduced into the two-stage absorption tower, then, after desulfurization, it is introduced into the chimney through the high pressure dust catcher. The flue-gas desulfurization system consists of SO_2_ absorption system, flue-gas system, absorbent preparation system, gypsum treatment system, processed water system and accident slurry system. The whole system is very complex and involves many candidate variables that we do not know whether they are related to the output variable or not. There all 52 input variables provided by factory’s DCS. Table A1 presents the name, description and unit of these variables. In fact, there are too many variables in the candidate variables pool that may cause modeling inaccuracies. Meanwhile, it is very difficult to precisely select variables and model for such a complex process simply through domain knowledge. Therefore, the variable selection algorithm based on statistical theory is very meaningful to the accurate modeling of the process.

The dataset from the power plant is entered into the three algorithms used in Section 4, 50% of the dataset is used for modeling, and 50% of the dataset is used to test the accuracy of the model. Table 3 shows the modeling accuracy.

**Figure 9:**
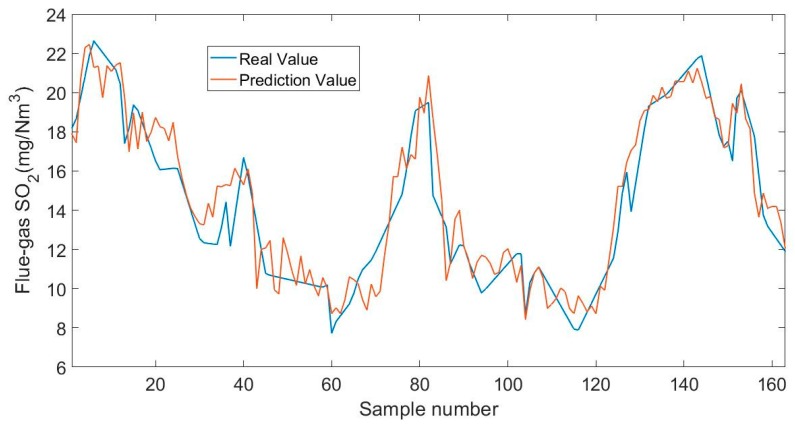
Measured and the predicted flue gas SO_2_ concentration.

**Figure 10:**
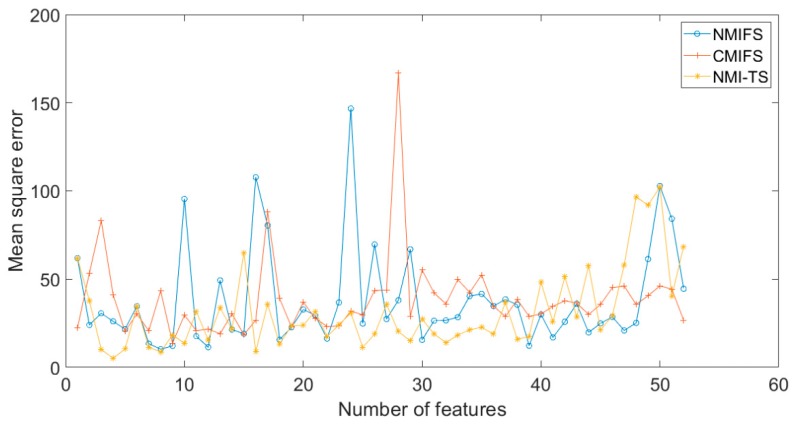
The curves of MSE number of features of the practical example with MI-based algorithms.

The soft sensor presented in this paper achieved the highest accuracy in predicting the SO_2_ concentration. Some data was input into the soft sensor based on NMI-TS, and the predicted value was compared with the real value, as shown in Figure 9. Figure 10 presents the curves of MSE to the number of features of desulfurization process with MI-based algorithms. The best solution appears at the point of four input variables. At the left of the point, the MSE deteriorates drastically as the number of variables decreases. Similar to Figure 2 and Figure 6, the NMI-TS has the best convergence rate among these algorithms. But unlike Figure 2 and Figure 6, the MSE tends to increase with fluctuating changes at the right of the point. The reason for this phenomenon is that the practical dataset has large cross-correlation between different input variables as well as uncertainty between redundant and useful information.

According to statistical results, there are four input variables selected by NMI-TS in the optimal solution, shown as Table 4.

The first variable is variable 3 that is the current of the third circulating slurry pump. There are four pumps in the tower 9-2, and every pump has its own motor to adjust the spraying speed of the slurry to control the rate of absorption. Usually, the operator takes the default operation mode under which there are two pumps normally open in the tower 9-2, and manually adjusts the switch and frequency of the third motors according to the production situation. Therefore, variable 3 is highly related to the target variable and included in the optimal solution. Secondly, variable 33 is the outlet’s flue gas SO_2_ concentration of the first tower and the inlet’s flue gas SO_2_ concentration of the second tower, which is obviously correlated to the final SO_2_ concentration. The third one is variable 38 that is the limestone slurry to the absorption towers. It can be seen from reaction Formulaes (8) and (9) that the degree of this reaction is closely related to the amount of CaO and ${\mathrm{CaCO}}_{3}$, i.e., the limestone slurry. The fourth one is variable 50 that is the pH value of the slurry in the tower 9-2. This result is quite consistent with basic chemical reaction mechanism and operational experience in the field. If the SO_2_ concentration of flue gas is relatively high, the SO_2_ absorbed by the slurry will be relatively more and therefore lower the pH value of the slurry. Conversely, a relatively lower SO_2_ concentration results in a higher pH value of the slurry.

## 6. Conclusions

The proposed algorithm for soft sensors is an innovative combination of NMI and TS. NMI-TS makes use of model accurateness to guide the process of variable selection by the NMI algorithm, and avoids the local optimal situation by using TS. We use simulation examples with different scales, correlations, and noise intensities to verify the superiority of our algorithm. Furthermore, the NMI-TS soft sensor was applied to a power plant desulfurization process. The simulation results show that the NMI-TS soft sensor can achieve high prediction accuracy with a simpler model of SO_2_ concentration. The proposed soft sensor is very easily implemented and adaptable since the program can be stored as a subroutine in the host computer of DCS. With the huge amount of production data newly provided by the DCS, the soft sensor model could be easily retrained and updated by calling the subroutine at regular intervals.

In future research, it would be of interest to develop distributed parallel framework [15,16] of the proposed algorithm to solve the problem of increased computational complexity created by big data. The idea of distributed parallel framework could be combined with the proposed algorithm to obtain better and faster feature selection methods that may be successfully applied to large databases.

## Figures and Tables

**Table 1:** Simulation results of dataset.

	M.S	MSE	$R^{2}$	WS+	WS−
ANN	20	0.1895	0.8747	N/A	N/A
NNG-ANN	13	0.1616	0.8960	10.0%/1	30.7%/4
NMIFS	16	0.1842	0.8781	10.0%/1	43.7%/7
CMIFS	17	0.1617	0.8941	0	41.2%/7
NMI-TS	11	0.1534	0.9003	0	9.1%/1

**Table 2:** Simulation results of Friedman dataset.

	M.S	MSE	$R^{2}$	WS+	WS-
ANN	50	3.8992	0.9216	N/A	N/A
NNG-ANN	20	2.0691	0.9598	20.0%/1	80.0%/16
NMIFS	6	1.5943	0.9687	100.0%/5	100.0%/5
CMIFS	7	1.4257	0.9723	60.0%/3	71.4%/5
NMI-TS	6	1.2076	0.9764	20.0%/1	33.3%/2

**Table 3:** Simulation results of power plant dataset.

	M.S	MSE	$R^{2}$
ANN	52	23.4420	0.5124
NNG-ANN	10	8.8661	0.8243
NMIFS	8	10.2379	0.7710
CMIFS	9	13.6934	0.7225
NMI-TS	4	5.1309	0.8272

**Table 4:** Variables selected by normalized mutual information-tabu search (NMI-TS) in the optimal solution.

Number	Input Variable	Units
3	#9-3 Circulating SP current	A
33	#9-1AT outlet’s flue gas SO_2_	mg/Nm^3^
38	Limestone slurry to #9 AT flow	Nm^3^/h
50	#9-2AT’s pH value 1	

## Appendix A

**Table A1:** All input variables in distributed control systems (DCS).

Number	Input Variable	Units
1	#9-1 Circulating SP current	A
2	#9-2 Circulating SP current	A
3	#9-3 Circulating SP current	A
4	#9-4 Circulating SP current	A
5	#9Cycle SP E B phase current	A
6	#9Cycle SP G B phase current	A
7	AT gypsum DP 2′s current	A
8	AT gypsum DP 1′s current	A
9	Gypsum slurry DP A’s current	A
10	Water ring vacuum pump’s current	A
11	#9AT’s inlet flue-gas temperature	K
12	#9AT’s outlet flue-gas temp.1	K
13	#9AT’s outlet flue-gas temp. 2	K
14	#9AT’s outlet flue-gas temp.3	K
15	#9-2 AT outlet temperature 1	K
16	#9-2 AT outlet temperature 2	K
17	#9 AT’s gypsum slurry density	kg/m^3^
18	Limestone slurry density	kg/m^3^
19	#9 Furnace chimney inlet net flue-gas pressure	kPa
20	#9-2 AT outlet pressure	kPa
21	AT gypsum DP 1′s inlet pressure	kPa
22	AT gypsum DP 2′s inlet pressure	kPa
23	#9 Gypsum slurry cyclone’s inlet pressure	kPa
24	Gypsum SP to #9 AT tube’s pressure	kPa
25	Generator Power	kW
26	#9 Absorber liquid level’s value	m
27	#9-2 AT liquid level 1	m
28	#9-2 AT liquid level 2	m
29	#9-2 AT liquid level 3	m
30	Gypsum slurry tank’s level	m
31	Belt dewatering machine A’s gypsum filter cake thickness	m
32	#9Furnace’s original flue-gas SO_2_	mg/Nm^3^
33	#9-1AT outlet’s flue-gas SO_2_	mg/Nm^3^
34	#9 Furnace original concentration of flue-gas and dust	mg/Nm^3^
35	#9 Furnace original flue-gas NOx	mg/Nm^3^
36	#9 Furnace original flue-gas O_2_	mg/Nm^3^
37	#9-1 AT outlet flue-gas O_2_	mg/Nm^3^
38	Limestone slurry to #9 AT flow	Nm^3^/h
39	#9-2AT’s feed flow	Nm^3^/h
40	Total air volume	Nm^3^/h
41	Total coal	Nm^3^/h
42	#9Furnace’s chimney inlet net flue-gas flow	Nm^3^/h
43	#9 original smoke flow	Nm^3^/h
44	#9 AT gypsum’s slurry flow	Nm^3^/h
45	AT gypsum DP 1′s speed feedback	r/s
46	AT gypsum DP 2′s speed feedback	r/s
47	Gypsum SP A’s speed feedback	r/s
48	Vacuum belt dewatering machine’s speed feedback	r/s
49	#9 AT gypsum’s slurry pH value	
50	#9-2AT’s pH value 1	
51	Unit 9 desulfurization efficiency	
52	Intermediate value of desulfurization efficiency of Unit 9	

AT = Absorption tower; DP = discharge pump; SP = slurry pump.

## References

[1] Paulsson D., Gustavsson R., Mandenius C.F. (2014). A soft sensor for bioprocess control based on sequential filtering of metabolic heat signals. Sensors.

[2] Chen K., Liang Y., Gao Z., Liu Y. (2017). Just-in-time correntropy soft sensor with noisy data for industrial silicon content prediction. Sensors.

[3] Kaneko H., Arakawa M., Funatsu K. (2010). Development of a new soft sensor method using independent component analysis and partial least squares. Aiche J..

[4] Ahmed F., Nazir S., Yeo Y.K. (2009). A recursive PLS-based soft sensor for prediction of the melt index during grade change operations in HDPE plant. Korean J. Chem. Eng..

[5] Wang D., Liu J., Srinivasan R. (2010). Data-driven soft sensor approach for quality prediction in a refining process. IEEE Trans. Ind. Inform..

[6] Xing H., Jun J., Kaixin L., Zengliang G., Yi L. (2019). Soft sensing of silicon content via bagging local semi-supervised models. Sensors.

[7] Yuan X., Huang B., Wang Y., Yang C., Gui W. (2018). Deep learning-based feature representation and its application for soft sensor modeling with variable-wise weighted SAE. IEEE Trans. Ind. Inform..

[8] Mambou S., Krejcar O., Kuca K., Selamat A. (2018). Novel cross-view human action model recognition based on the powerful view-invariant features technique. Future Internet.

[9] Mambou S., Krejcar O., Maresova P., Selamat A., Kuca K. (2019). Novel hand gesture alert system. Appl. Sci..

[10] Feil B., Abonyi J., Pach P., Nemeth S., Arva P., Nemeth M., Nagy G. (2004). Semi-mechanistic models for state-estimation–soft sensor for polymer melt index prediction. Artificial Intelligence and Soft Computing.

[11] Ko Y.D., Shang H. (2011). A neural network-based soft sensor for particle size distribution using image analysis. Powder Technol..

[12] Shakil M., Elshafei M., Habib M.A., Maleki F.A. (2009). Soft sensor for NO_x_ and O_2_ using dynamic neural networks. Comput. Electr. Eng..

[13] Bo C.M., Li J., Sun C.Y., Wang Y.R. The application of neural network soft sensor technology to an advanced control system of distillation operation. Proceedings of the International Joint Conference on Neural Networks.

[14] Jiesheng W., Shuang H., Nana S. (2014). Features extraction of flotation froth images and BP neural network soft-sensor model of concentrate grade optimized by shuffled cuckoo searching algorithm. Sci. World J..

[15] Le Y., Zhiqiang G. (2018). Big data quality prediction in the process industry: A distributed parallel modeling framework. J. Process. Control.

[16] Xinyu Z., Zhiqiang G. (2019). Local parameter optimization of LSSVM for industrial soft sensing with big data and cloud implementation. IEEE Trans. Ind. Inform..

[17] Greenland S. (1989). Modeling and variable selection in epidemiologic analysis. Am. J. Public Health.

[18] Hui Z., Hastie T. (2005). Addendum: Regularization and variable selection via the elastic net. J. R. Stat. Soc..

[19] Raftery A.E., Dean N. (2017). Variable selection for model-based clustering. J. Am. Stat. Assoc..

[20] Wang J.L., Zhang J., Wang X.X. (2018). A data driven cycle time prediction with feature selection in a semiconductor wafer fabrication system. IEEE Trans. Semicond. Manuf..

[21] Mcnitt-Gray M.F., Huang H.K., Sayre J.W. (1995). Feature selection in the pattern classification problem of digital chest radiograph segmentation. IEEE Trans. Med. Imaging.

[22] Le Y., Zhiqiang G. (2018). Variable selection for nonlinear soft sensor development with enhanced binary differential evolution algorithm. Control Eng. Pract..

[23] Zhiqiang G., Zhihuan S. (2010). A comparative study of just-in-time-learning based methods for online soft sensor modeling. Chemom. Intell. Lab. Syst..

[24] Sun K., Liu J., Kang J.L., Jang S.S., Wong S.H., Chen D.S. (2014). Development of a variable selection method for soft sensor using artificial neural network and nonnegative garrote. J. Process Control.

[25] Frénay B., Doquire G., Verleysen M. (2013). Is mutual information adequate for feature selection in regression?. Neural Netw..

[26] Battiti R. (1994). Using mutual information for selecting features in supervised neural net learning. IEEE Trans. Neural Netw..

[27] Soofi E.S., Retzer J.J. (2003). Information importance of explanatory variables. IEE Conference in Honor of Arnold Zellner: Recent Developments in the Theory, Method and Application of Entropy Econometrics.

[28] Hanchuan P., Fuhui L., Chris D. (2005). Feature selection based on mutual information: Criteria of max-dependency, max-relevance, and min-redundancy. IEEE Trans. Pattern Anal. Mach. Intell..

[29] Estévez P.A., Michel T., Perez C.A., Zurada J.M. (2009). Normalized mutual information feature selection. IEEE Trans. Neural Netw..

[30] Bennasar M., Hicks Y., Setchi R. (2015). Feature selection using joint mutual information maximisation. Expert Syst. Appl..

[31] Novovicová J., Somol P., Haindl M., Pudil P. (2007). Conditional mutual information based feature selection for classification task. Iberoamerican Congress on Pattern Recognition.

[32] Zhang H., Sun G. (2002). Feature selection using tabu search method. Pattern Recognit..

[33] Pachecoaab J. (2009). A variable selection method based on tabu search for logistic regression models. Eur. J. Oper. Res..

[34] Osman I.H. (1993). Metastrategy simulated annealing and tabu search algorithms for the vehicle routing problem. Ann. Oper. Res..

[35] Lin W.M., Cheng F.S., Tsay M.T. (2007). An improved tabu search for economic dispatch with multiple minima. IEEE Power Eng. Rev..

[36] Enrique R., Josep María S. (2008). Performing feature selection with multilayer perceptrons. IEEE Trans. Neural Netw..

[37] Peterson L.E. (2009). K-nearest neighbor. Scholarpedia.

[38] Kraskov A., Stögbauer H., Grassberger P. (2004). Estimating mutual information. Phys. Rev. E Stat. Nonlinear Soft Matter Phys..

[39] Fukunaga K. (2013). Introduction to Statistical Pattern Recognition.

[40] Khaksar W., Hong T.S., Khaksar M., Motlagh O. (2014). A fuzzy-tabu real time controller for sampling-based motion planning in unknown environment. Appl. Intell..

[41] Hongxing W., Wang B., Wang Y., Shao Z., Chan K.C.C. (2012). Staying-alive path planning with energy optimization for mobile robots. Expert Syst. Appl..

[42] Faris H., Aljarah I., Mirjalili S. (2017). Improved monarch butterfly optimization for unconstrained global search and neural network training. Appl. Intell..

[43] Pan C.C., Bai J., Yang G.K., Wong S.H., Jang S.S. (2012). An inferential modeling method using enumerative PLS based nonnegative garrote regression. J. Process Control.

[44] Friedman J.H. (1991). Multivariate adaptive regression splines. Ann. Stat..

